# Testing the utility of the first step of system evaluation theory in creating a system map of care for cardiac amyloidosis early detection: A case study

**DOI:** 10.1371/journal.pone.0339063

**Published:** 2026-01-16

**Authors:** Sherry L. Ball, Alexis Koskan, Jenice Guzman, Sandesh Dev

**Affiliations:** 1 Research Service, VA Northeast Ohio Healthcare System, Cleveland, Ohio, United States of America; 2 College of Health Solutions, Arizona State University, Phoenix, Arizona, United States of America; 3 Southern Arizona VA Health Care System, Tucson, Arizona, United States of America; Sarich Neuroscience Research Institute, AUSTRALIA

## Abstract

**Background:**

Heart failure is a clinical syndrome resulting from numerous pathological conditions. One cause of heart failure, transthyretin cardiac amyloidosis, presents insidiously with common and seemingly unrelated symptoms. New treatments for cardiac amyloidosis are available that extend and improve life. However, providers are not testing patients for transthyretin cardiac amyloidosis. We took a systems science approach to explore the system of care for transthyretin cardiac amyloidosis testing by depicting the healthcare system from patient presentation to treatment. Our goal was to define an ideal healthcare system to improve the uptake of testing protocols and enhance patient outcomes.

**Methods:**

We assembled clinicians, researchers, and patients to participate in a co-design workshop using the first step of System Evaluation Theory to define an ideal testing and diagnostic protocol using transthyretin cardiac amyloidosis as a case study. We tasked workshop attendees with defining the patient and clinician journey from symptom presentation to diagnosis. We generated a system map using a qualitative matrix analysis of a transcript of the workshop discussion.

**Results:**

The matrix analysis organized input from all stakeholders, allowing for the creation of a system map that reveals the complexity of the transthyretin cardiac amyloidosis testing process and potential implementation strategies to improve the efficiency and effectiveness of the system. This methodology successfully yielded generalizable elements of a testing protocol and testable strategies to facilitate the implementation of a protocol adapted to fit local site needs.

**Conclusions:**

The substeps outlined within System Evaluation Theory Step 1 helped identify an ideal system for testing and diagnosing transthyretin cardiac amyloidosis care that could be applied to specific settings to identify, improve, and implement protocols for other complex diseases.

## Introduction

Although implementing evidence-based guidelines to diagnose and treat medical conditions can prolong life, they are often underutilized [1]. While reasons for guideline underutilization for individual medical conditions vary, a challenge for many chronic conditions lies in the need for coordination between primary care providers and clinicians with a wide range of specialized expertise [2]. In these cases, successful guideline implementation often depends on individual, clinical, departmental, and organizational-level interactions [3,4]. These multi-level influences can make defining implementation steps perplexing [5]. Thus, a methodological protocol for identifying potential processes for coordinating and integrating specialized knowledge could be the first step toward improving guideline implementation. Our goal was to test and adapt an existing protocol for mapping a complex system. As a case study, we chose to map a multidisciplinary system of healthcare for earlier diagnosis of an under-recognized complex chronic condition, cardiac amyloidosis (CA) [6,7]. Cardiac amyloidosis results from misfolding of proteins that then collects within the heart muscles as amyloid fibrils and causes cardiac structural and functional impairments [8]. There are two major types of proteins often associated with cardiac amyloidosis – monoclonal immunoglobulin light change (AL) and transthyretin (ATTR) which could be a wild-type (often associated with aging) or due to a genetic mutation (variant) [8]. Because transthyretin proteins can settle in other tissues, clinical manifestations may include musculoskeletal symptoms, polyneuropathy and autonomic dysfunction [8]. Early diagnosis and treatment are preferred to prevent progression of the disease, improve quality of life and increase survival [9].

Diagnosing ATTR-CA in patients requires coordination and communication among multiple healthcare specialties. Due to the systemic manifestation of this condition, we chose to examine the diagnosis of ATTR-CA as a complex system. We define a complex system as one composed of multiple healthcare professionals (system parts) interacting in response to a range of healthcare data (system inputs) that must be considered as a whole to reach a diagnosis. We propose using System Evaluation Theory (SET) [5,8,10] as a framework to develop a clinical workflow that includes three steps. The first step identifies all stakeholders; the second optimizes awareness and communication among specialists for the timely diagnosis of ATTR-CA (particularly bone scintigraphy with monoclonal protein testing), and the third examines these workflows to identify implementation strategies to promote current evidence-based clinical guidelines to detect ATTR-CA [6].

Compared to other frameworks, SET is specifically designed to define and evaluate complex interventions such as the diagnosis of ATTR-CA which depends on multiple interacting healthcare components thus meeting the criteria of a system [7,8]. Other similar mapping methodologies, such as patient journey/experience maps [11], focus on the customer or patient experience. Although our system map begins with the patient, our goal was to test whether the SET methodology could provide an ideal template that may be used to guide a healthcare system in implementing a multidisciplinary system for the early diagnosis of ATTR-CA. While service blueprints [12] and workflow process mapping [13] are similar to SET Step 1, we found the SET more accessible. SET uses a systematic approach to define and evaluate complex interventions as a system with a defined boundary of interdependent parts, feedback loops, and/or reflex arcs that generate an emergent property [5,8]. Because SET is aligned with operational and functional ATTR-CA design characteristics, it is the best fit for this work.

SET Step 1 (composed of multiple subsets) is focused on engaging stakeholders to define the system parts and connections, including the boundaries of the ATTR-CA system. The ATTR-CA boundaries are defined by obtaining input from program participants about related information technology infrastructure, resources, and auxiliary influences [5,8]. Hereinafter, we refer to the defined ATTR-CA system boundaries, components, and their interactions collectively as a system map. This first step defines the ATTR-CA system parts, establishes their connections, and details these connections in the form of joint standard operating procedures. Our work was centered on applying SET Step 1.

SET Step 2 focuses on defining ATTR-CA interdependencies. SET’s focus on interdependencies is one critical feature that differentiates it from other frameworks. Where components interact is the most likely place the system will experience inefficiencies [8]. Other quality improvement approaches do not focus on system interdependencies. Given the reality of finite evaluation resources and the evaluation capacity of most agencies, SET’s methodology helps define *precisely* where and how these interactions occur. It uses a series of system concepts (e.g., feedback loops, reflex arcs, cascading events) [5] to monitor for inefficiencies. Pinpointing areas of inefficiency is critical in any health system, but especially for the system of care for the diagnosis of ATTR-CA which has life-and-death consequences.

Step three of SET defines and evaluates the system’s emergent property. This is another key feature that differentiates SET from other frameworks. Many frameworks and analyses (e.g., multiple regression) take a siloed approach, defining and evaluating the independent contributions of each system part [5]. For ATTR-CA testing and diagnosis (and many other health systems), this is a nonsensical approach because whether lives are saved depends on the successful coordination of all ATTR-CA system components [8]. No single health system component can independently save a life. Rather, each part depends on the other to meet the system’s goal. By extension, this dependence means that the failure of any one part can lead to a complete system failure. SET focuses on measures of effectiveness outcomes that make sense given the system’s functional purpose [5].

We describe how we used the subsets of SET Step 1 to define the ATTR-CA system and identify associated implementation strategies. More specifically, this paper describes lessons learned using SET Step 1 as a framework. We designed and conducted a workshop and follow-up interviews where stakeholders (subject matter experts) were guided through a systems-thinking protocol to develop an evidence-based system map for early testing for ATTR-CA and to identify implementation strategies to facilitate adherence to the map. We discuss how we conducted the workshop and followed SET Step 1 to define a complex system [5], beginning with identifying workshop participants, collecting critical information before the workshop, conducting the in-person workshop, setting realistic workshop goals, analyzing data, interpreting the findings, completing member checking, and ending with a system map.

## Methods

### Setting

#### Conference.

The study’s last author led the planning of a one-day conference titled *“*Updates in Cardiac Amyloidosis,” held on January 14, 2023. Clinicians, many of whom were also researchers, with expertise in testing, diagnosing, and treating cardiac amyloidosis were invited to present on related topics. The conference focused on state-of-the-art research relevant to the field.

#### Post-conference workshop.

Our research team scheduled a three-hour systems-mapping workshop immediately following the conference. We applied the methods outlined in the substeps of SET Step 1 in this case study. We sought to determine the extent to which SET could be used to identify the system of care.

#### Workshop participants.

We used purposive sampling from the list of conference attendees to include representation from each medical discipline involved in identification, testing, and/or diagnosis. We invited these 12 attendees to the post-conference workshop via email. Additionally, we invited two local cardiac amyloidosis patients (treated and recruited by workshop attendee clinicians), one with ATTR-CA and one with another form of CA (AL (light chain)-CA), to incorporate the patient perspective in creating this system map.

All workshop participants completed an informed consent form, which included permission to audio-record the session. The workshop proceedings were transcribed to capture the discussions and input from participants. All participants were offered $100 as remuneration for their shared expertise and time. The Arizona State University Institutional Review Board approved the conduct of this work.

*Pre-workshop questionnaire*: We used methods described in SET Step 1, Substeps I and II to define the intervention. We designed a pre-workshop questionnaire (S1 File) and distributed pre-workshop questionnaire online using Qualtrics to all workshop invitees. The questionnaire included open-ended questions designed to generate an exhaustive list of individuals, resources, information, and processes related to testing for and diagnosing ATTR-CA, as well as any related barriers and facilitators. Responses from the pre-workshop questionnaire were distributed at the start of the workshop and used to generate further discussion.

*Workshop discussion guide*: The workshop followed a discussion guide (S2 File) developed based on the SET framework to generate data that informed the creation of a system map depicting the patient journey from symptom presentation to diagnosis. Our discussion guide focused on identifying stakeholders and their roles, knowledge, and skills related to ATTR-CA; resources provided and/or needed by each stakeholder; where and how needed information is communicated between stakeholders; and how the contribution of each stakeholder impacts and is impacted by other stakeholders.

*Data analysis:* We organized data collected directly from the workshop transcript using a matrix analysis approach [11].

*Member checking*: Using a follow-up interview guide (S3 File), we conducted follow-up interviews with workshop attendees via Microsoft Teams. A draft system map was disseminated to workshop participants and adjusted based on their feedback to create a final standard operating procedure to test for ATTR-CA in a clinical setting. Due to the scheduling challenges of convening the workshop participants for an additional joint meeting, we used an individual member-checking exercise. Specifically, we invited all clinician workshop participants to share their availability for a one-hour virtual follow-up interview, during which they were asked to provide feedback on the accuracy of the draft systems map, recommend changes, and suggest implementation strategies to improve the timely diagnostic work-up for ATTR-CA. Additional stakeholders (e.g., one sonographer and two cardiologists) who could not attend the January workshop but were identified as healthcare providers with extensive experience working with ATTR-CA patients were added to the list of invited participants.

## Results

### SET Step 1: Define the complex intervention as a system

#### SET Step 1 Substeps I and II: Get leaders to the table and ensure buy-in and define the emergent system property: *Pre-workshop participant engagement and planning.*

*Pre-workshop questionnaire*: Ten workshop participants completed the online questionnaire. Responses were compiled and summarized (Table 1). A compilation of all responses is included for each topic. Responses from the questionnaire helped guide the discussion about barriers and facilitators to ATTR-CA workup. We hosted 10 workshop participants, including eight clinicians experienced in the care of patients with cardiac amyloidosis who represented various medical specialties (hematology, heart failure cardiology, neurology, pathology, genetic counseling, primary care, nuclear radiology) and two patients with cardiac.

**Table 1:** Results of Qualtrics survey about the early detection of ATTR-CA.

Results of the Qualtrics Survey
**Topic 1: Regarding the early diagnosis of cardiac amyloidosis:** **Who is involved? What is their role? What resources and/or knowledge are needed?**
Community Members
• Patient, patient’s caregiver
• Family members who have the genetic disease
• Need to alert the family to see a cardiologist
Healthcare Professionals involved
Primary care/Internists: Increased awareness of the condition
• Increased awareness of the disease
• Perception that amyloidosis is not a rare disease or condition
• Appropriate referrals to prompt genetic testing, relationship with genetic testing
labs
• Identify the need to see a cardiologist
• Comfort with the testing paradigm
Genetic testing counselors -single test for hereditary ATTR amyloidosis
Cardiac sonographer – echocardiography – use an echocardiogram to detect AL amyloidosis
• Need proper training and understanding of cardiac amyloidosis
• Radiologist – use of nuclear imaging for detection of amyloidosis
• Pathologist – clinical indications for biopsy provided by EMR are often not accurate, so targeted amyloid testing by indication could be challenging – they need additional information
• Pulmonologist – sees patient with shortness of breath
• Nephrologist (AL) orders the appropriate tests for CA
• Need access to expert renal pathologists
• Must be comfortable obtaining a renal biopsy
• Neurologist (AL and ATTR) – think about diagnosis (a disorder of peripheral nerves) and order appropriate testing
• Need access to fat aspirate for amyloid tissue dx outside specialty centers
• Hematologist & Oncologist – assess whether the patient with AL cardiac amyloidosis is a candidate for chemotherapy
• Must be aware of amyloidosis
• MGUS follow-up/initial evaluations often focus on myeloma symptoms.
• Less attuned to asking patients about the shortness of breath and any swelling that may occur (due to CA)
Orthopedic surgeon – may see symptoms such as carpal tunnel syndrome, trigger finger
Cardiologists
• Need input from a pulmonologist
**Topic 2: What are the barriers to the early diagnosis of cardiac amyloidosis?**
Community Members and Providers
• Lack of awareness of the disease and associated symptoms
• For providers, amyloid can be a great mimicker of other diseases
• Access to healthcare, specifically healthcare system with access to MRI, PET, biopsy, labs, cardiologist
Healthcare Professionals
• Lack of referring provider access
• Lack of awareness of genetic counseling resources/services
• Lack of provider training in cardiac amyloidosis
• Lack of proper algorithm for diagnosis
• Lack of proper interpretation of cardiac PYP scan
• Treating patients for AL amyloid instead of ATTR – because mass spectroscopy was not used as part of the diagnostic workup
• Providers do not consider cardiac amyloidosis in their differential diagnosis
• Doctors not communicating
• Provider attitude of being too busy
• Provider lack of time
• Provider apathy
**Topic 3: What are the facilitatorsto increasing early diagnosis of cardiac amyloidosis?**
Community Members
• Spokesperson, cardiac amyloidosis champion
• Access to amyloid centers of excellence for expedited care from multidisciplinary teams
Healthcare Professionals
• Improved awareness among providers that amyloidosis is more prevalent than what they previously assumed
• Case studies of patients with CA
• Patient reports
• How the diseases changed their lives
• Reports of misdiagnosis, late diagnosis – when an underlying health issue is
cardiac amyloidosis
• Algorithms to better predict the disease
• Improved lab testing for amyloid subtyping via mass spectrometry
• Improved imaging
• History and physical followed by an echocardiogram with strain, EKG, and cardiac biomarkers
• Adequate funding for programs to increase awareness about cardiac amyloidosis
• Training in educational settings allows students training to be healthcare providers to see what the disease looks like in real life versus textbooks and video.

The team divided the participants into two breakout groups and discussed the questionnaire results. Responses were distributed at the start of the workshop and used to generate further discussion. Participants made edits to the results, adding, removing, and revising the list of barriers and facilitators to the early detection of ATTR-CA. One person from each group reported a summary of their group’s discussion.

*Workshop discussion:* The team sent the audio-recorded discussion for transcription to facilitate analysis of the discussion results. The transcript was used to populate a matrix, later used in the development of the draft system map of care for ATTR-CA. The team created a matrix to organize the various components of a system map of care for ATTR-CA. In the matrix, rows identified who performed different roles in the early diagnosis system of care (e.g., clinician, patient, caregiver), and columns identified tasks, procedures, needs (e.g., revenue, time, expertise), and resources needed for the early detection of CA. Table 2 operationalizes the following identified matrix column headers: role (CA testing related tasks, education/awareness, credentials/reputation), resources needed by row item (expertise/knowledge, clinical tests/procedures, time, revenue, other tools, EMR); resource provided by (expertise/knowledge, clinical tests/procedures, time, other tools, EMR); attitudes/mindset; awareness, communication (what, how, to/from), costs, and marketing/outreach. Each row included column information related to one identified individual role (e.g., patients and clinicians) in the system. We identified how different individuals, resources, and other map components intersected. We took an iterative approach to refine the matrix by reviewing and redefining column headers, clarifying the meaning by reviewing the workshop transcription, and gaining the research team’s consensus on the matrix. The complete set of data populate in the matrix is included in S4 File.

**Table 2:** Matrix analysis components column headers and the operational definitions used to populate the matrix analysis table are included.

Domain	Operational definition and/or Subdomain
**Role**	Actions performed by individual person/role that impact CA screening
	Credentials/reputation reflective of impact on CA screening
**Resources needed by row item**	What is known by individual that impacts CA screening
	Clinical tests/procedures
	Equipment
	Time
	Revenue
	Other tools, anything used in CA screening
	Electronic medical record (EMR)
**Resource provided by row item**	Expertise/knowledge what is known by this individual that impacts CA screening
	Clinical tests/procedures
	Equipment
	Time
	Other tools, anything used in CA screening
	EMR
**Attitudes**	Prior thoughts or beliefs about the need for CA screening
**Awareness**	Any prior knowledge about CA screening
**Skills/knowledge**	Existing or needed knowledge or screens related to CA screening
**Communication mechanism**	What is communicated
	How communication happens
	Who or where information is and where it goes
**Financial costs**	Expenditure of funds
**Marketing/** **outreach**	Materials intended to inform patients and clinicians about CA screening
**Recommendations**	Suggestions and/or ideas for improvement
**Efficiency**	Ratio of benefit to required resources

#### SET Step 1 Substep III: Define the system parts, the ATTR-CA components.

*Matrix analysis:* To complete substep III, we used the populated matrix to identify and map individual parts of the system (e.g., who is involved, resources, data, and actions needed, etc.) and created a draft of the system map depicting the patient and provider journey through the testing process. This process allowed us to identify the system of care components (people, knowledge, and resources) and current clinical practices needed to identify and test patients for ATTR-CA, examine the interactions between these components, and identify implementation strategies for the timely workup of cardiac amyloidosis.

#### SET Step 1 Substep IV: Connect the parts.

**Fig 1:**
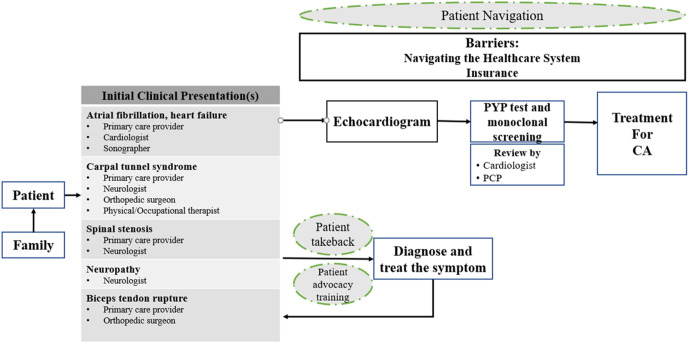
Draft systems map of the early diagnosis of cardiac amyloidosis. The systems map begins on the left-hand side with the patient presenting to a PCP or specialist any of a list of clinical symptoms followed by tests reviewed by a cardiologist and a treatment decision is made. During the process, patients may be influenced by family, a patient advocate, and/or a patient navigator. Black arrows = Patients’ potential steps to receiving care. Green dashed circles = Implementation strategies. Abbreviations: PYP = pyrophoshate;PCP = primary care; CA = cardiac amyloidosis.

*System Mapping*: Using the matrix, one research team member created a draft ATTR-CA system map. Our initial system map (Fig 1) began with the patient, possibly influenced by a family member, presenting early symptoms of ATTR-CA to a range of potential clinicians, usually leading to one of two paths (bottom half of figure): 1) patients are diagnosed and treated based on individual symptoms; or 2) patients receive follow-up testing such as an echocardiogram, PYP test, and monoclonal screening that is reviewed for possible ATTR-CA by a cardiologist before treatment for ATTR-CA. Noted barriers indicated at the top of the map included navigating the healthcare system or a lack of insurance coverage. Potential interventions that could facilitate patients receiving comprehensive screening for ATTR-CA included patients learning to advocate for themselves with their clinicians or having assistance from a patient navigator to manage any barriers encountered while undergoing often multiple tests and seeing numerous specialists.

The resulting draft system map and explanatory video (Fig 1 along with a link to a seven-minute video describing the draft systems map for the early detection of CA; link: https://youtu.be/i74K78BmRX0) was emailed to all workshop participants.

#### SET Step 1 Substep V: Use the identified systems map to develop operating procedures.

**Fig 2:**
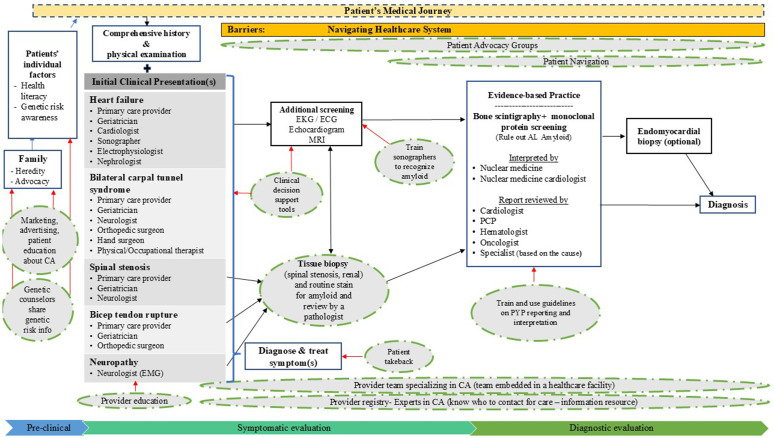
Revised system map for the early detection of CA. System map for the early detection of CA/ Black arrows = Patients’ potential steps to receiving care; Green dashed circles = Implementation strategies; Red arrows = Implementation strategy influencing a patient stage; Blue arrows = Influence on patient stage. Abbreviations: EKG/ ECG = electrocardiogram, MRI = magnetic resonance imaging, AL amyloid = amyloid light chain, PCP = primary care provider.

*Member checking*: To assess participants’ perceptions of the draft map and to expand or change it as needed, we conducted member-checking interviews with workshop participants. On average, member-checking interviews lasted one hour. After the interviews, all transcripts were reviewed against the distributed draft version of the systems map and modified as needed based on participants’ recommendations. We employed an iterative process, where the same results or recommendations were reviewed and updated multiple times until we reached a consensus on the final version of the map (Fig 2). This version was then validated using the ATTR-CA literature.

Based on participants’ feedback on the original map, for the final map presented in Fig 2, we categorized the testing and diagnostic process into three stages: preclinical stage, symptomatic evaluation, and diagnostic evaluation. Organizing the map based into these three stages helped facilitate discussion around the map and identified implementation strategies that emerged during the original panel discussion and member-checking interviews. The pre-clinical stage occurs before a patient seeks care for CA symptoms. The symptomatic evaluation stage involves an initial comprehensive history and physical examination, as well as potential cardiac screenings and diagnostic tests that may necessitate bone scintigraphy and monoclonal protein testing. The diagnostic evaluation stage encompasses bone scintigraphy and monoclonal protein testing, as well as potential downstream tests once the index of suspicion for cancer is raised. We identified challenges and implementation strategies for each stage to enhance care encircled by a green dotted line. Table 3 describes the differences between the draft and final system map.

**Table 3:** Updates between the draft and final systems map.

Draft map content area	Expansion in final system map
Patient	Listed patient characteristics that influence screening. These include patients’ awareness of CA or ATTR-CA, awareness of risk for the condition, understanding of risk to family members in case of hereditary ATTR-CA] that may influence screening and health literacy
Family	Identified how family influences patients’ willingness to undergo screening for ATTR-CA by 1) sharing genetic information about hereditary ATTR-CA and 2) advocating for patients to seek care
New: Comprehensive history & physical examination	Added the critical step of obtaining a comprehensive medical history with a physical examination that may help identify initial clinical presentations for ATTR-CA
Initial Clinical Presentations	Reorganized the list of symptoms by the leading red flags for ATTR-CA and expanded the list of providers who may diagnose and treat these individual symptoms
Echocardiogram	Renamed this “Additional Screening” and listed the various types of additional diagnostic tests that patients may receive
PYP scan and monoclonal screening	Clarified that this is the evidence-based practice and focus of new implementation strategies (to increase bone scintigraphy) Renamed to Bone scintigraphy + monoclonal protein testing with rationale for using this screening Added information on who interprets and reviews reports for this diagnostic work-up
New: Endomyocardial biopsy	Added this additional test that may be used to confirm ATTR-CA

The first column, draft content area, lists the part of the map where the revisions were made and the second column, expansion in final system map, explains what changes were made.

## Discussion

We sought to test the application of the first step of SET to engage stakeholders in defining and mapping the implementation of the diagnostic work-up for cardiac amyloidosis. SET Step 1 consists of many substeps. SET Step 1 Substep I*: Convening and engaging leaders.* We invited healthcare professionals from various disciplines and with the specific expertise required to diagnose patients with ATTR-CA; these leaders helped us plan the workshop and identify the core components that allowed for an efficient use of time during the convened workshop. SET Step 1 Substep II: *Defining the emergent system property*. We worked with our engaged leaders to identify the goal of the system map by defining boundaries and emergent properties of the system. We define the system’s goal as improving the early detection of ATTR-CA from the perspective of healthcare professionals. SET Step 1 Substeps III and IV: *Defining and connecting the system parts*. We identified the roles of each stakeholder involved, as well as any necessary resources and knowledge. We asked how information was shared or communicated between stakeholders and resources. SET Step 1 Substep V: *Defining the operating procedures.* This step was completed using member checking, bringing the system map to all stakeholders to identify missing information or areas where processes, roles, tasks, or procedures needed further clarification.

Due to the limited time available with the ATTR-CA experts, building the system map had to occur outside the convened meeting. We organized data (audio recording of the meeting) using a qualitative matrix methodology [14]. This methodology provided rigor for identifying system map components based on information shared by expert stakeholders during the convened meeting.

In collaboration with a panel of CA experts, we created a system map that represents the essential steps for testing and diagnosis of ATTR-CA. Group and individual input from all stakeholders revealed the complexity of the ATTR-CA diagnosing process and identified opportunities for improvement, including care coordination, the need for specialized expertise, and the development of clear communication strategies. Our deliverables included an ideal implementation map for ATTR-CA guidelines and a mapping protocol that could be applied to develop strategies for implementing evidence-based practices for other complex chronic conditions. Although the American College of Cardiology published a diagnostic algorithm for detecting cardiac amyloidosis [15], the algorithm lacks detail on who performs which duties leading up to the diagnosis of ATTR-CA and patient characteristics that influence the early detection of this condition, details that our final system map includes.

Our methodology of using the SET Step 1 substeps to partner with stakeholders to create an initial system map draft, followed by updating and expanding the original draft based on member checking. This system map can be used to identify testable implementation strategies that facilitate the implementation of a protocol tailored to local site needs. The SET process facilitated the definition and mapping of an ideal system for testing and diagnosis, identified barriers and facilitators to ATTR-CA care, and provided a comprehensive understanding of the testing protocol as a complex system.

We expected SET to facilitate identifying, improving, and implementing protocols for testing and treating complex diseases. While using this framework, we learned valuable lessons that may improve its use for mapping a system of care for complex diseases. First, pre-workshop planning helped facilitate participant recruitment and engagement and refined the workshop agenda. SET informed the design of the workshop questionnaire, workshop agenda/discussion guide, in addition to discussions with two leaders in the field of cardiac amyloidosis who were also speakers at the conference. In addition to engaging participants before the workshop, the pre-workshop questionnaire facilitated the ability of all voices to be heard equally and improved workshop efficiency by identifying parts of the system map.

Workshop discussions yielded valuable insights into patients’ journeys through the early detection of ATTR-CA and identified key areas for improvement. Next, workshop attendees highlighted the importance of building structure into the workshop to efficiently and effectively use participants’ time to generate an actionable product. We agreed to plan for an interactive workshop after a morning conference of primarily didactic instruction. Third, to analyze workshop proceedings, the matrix analysis offered an expeditious approach to evaluate complex, dynamic, and time-sensitive issues. Workshop implementation required identifying roles and positions of healthcare staff who have and will share knowledge and skills to implement evidence-based practices. Fourth, for workshop participants to provide meaningful feedback on the draft map, they must be aware of each other’s roles for effective communication and, ultimately, implementation. For each role, additional inputs can affect the effectiveness of information transfer, and the information transfer process itself can be affected by outside influence. Especially when working with busy professionals who have time constraints, it is helpful to inform the interviewees’ information about the topic and goals of the interview [16]. We expanded on this concept by creating a short video explaining the map to help clinicians to provide more reflective input [17] on the draft map. Sharing content as a short video with audio via publicly available websites reduced the cognitive load in consuming the material. A visual of the map with a brief explanation in video format provided a convenient, low-cost, and low-effort method to review the materials. Future implementation researchers can tailor this system map based on the realities of healthcare delivery at their site and select implementation strategies that will have the most impact on their organization’s early detection of ATTR-CA.

### Limitations

The identified barriers and implementation strategies may be challenging for some healthcare providers to operationalize. Maps of ATTR-CA diagnosis and treatment existed prior to our study. Since we were unaware of these protocols, they were not used to inform our discussion. Rather, we invited participants to help map the early detection of CA from their perspective. Our findings mirrored those of other efforts [12] and identified additional barriers and implementation strategies to promote the early detection of this condition. Another limitation is that clinician participants worked in multiple healthcare systems, which highlighted the need to adjust the map depending on context. This added complexity to their input while also enhancing the external validity of our system map. For example, providers represented high-resource healthcare facilities with experience regularly working with patients presenting with amyloid. Low-resource facilities may experience additional barriers not presented in this research. Future research should utilize this map and tailor it to a specific healthcare system before selecting implementation strategies that will have the most significant influence on enhancing the adoption of evidence-based practices, such as bone scintigraphy and monoclonal protein testing, for the early detection of ATTR-CA. Once the identified system map is applied in a clinical setting, SET Steps 2 and 3 can be applied to evaluate its efficiency and effectiveness. In SET Steps 2 and 3, efficiency is measured through exploration of interdependencies between components of the system map, and effectiveness is measured by identifying the emergence of the system goals.

## Conclusion

Applying a systems science lens to the early detection of CA may increase the likelihood of providing actionable changes to lead to the timely diagnostic work-up for this condition. Our findings highlight the effectiveness of using SET to develop a protocol for early diagnosis of a complex chronic condition such as cardiac amyloidosis and provide lessons learned that may be useful for other researchers mapping a system of care. SET provided us with a logical order to identify and engage stakeholders in discussion and to complete a series of specific tasks, which yielded a product, a system map. This map provided a visual which further stimulated discussion are focused our work on a clear product. Future work could test this or a similar product or system map to further identify the needed implementation strategies to facilitate improvements in patient care.

## Supporting information

S1 File Pre-workshop brainstorming questions. These questions were distributed to all workshop invitees to generate an exhaustive list of system components.
(DOCX)

S2 File Workshop discussion guide. List of questions and processes used during the workshop to generate components of the system map.
(DOCX)

S3 File Post-workshop interview guide. Questions asked of workshop participants who agreed to be interviewed to obtain their input on the completeness and accuracy of the post-workshop system map.
(DOCX)

S4 File Completed matrix. Matrix populated with data from workshop discussion transcript and used to create system map.
(XLSX)

## References

[1] Fischer F, Lange K, Klose K, Greiner W, Kraemer A. Barriers and strategies in guideline implementation-a scoping review. Healthc Basel Switz. 2016;4(3):36. 10.3390/healthcare4030036 PMC5041037 27417624

[2] Joo JY. Fragmented care and chronic illness patient outcomes: a systematic review. Nurs Open. 2023;10(6):3460–73. 36622952 10.1002/nop2.1607 PMC10170908

[3] Francke AL, Smit MC, de Veer AJE, Mistiaen P. Factors influencing the implementation of clinical guidelines for health care professionals: a systematic meta-review. BMC Med Inform Decis Mak. 2008;8:38. doi: 10.1186/1472-6947-8-38 18789150 PMC2551591

[4] Mickan S, Burls A, Glasziou P. Patterns of “leakage” in the utilisation of clinical guidelines: a systematic review. Postgrad Med J. 2011;87(1032):670–9. doi: 10.1136/pgmj.2010.116012 21715571 PMC3181428

[5] Renger R. System Evaluation Theory: A Blueprint for Practitioners Evaluationg Complex Interventions Operating and Functioning as Systems. Charlotte, NC: Information Age Publishing, Inc.; 2022. pp. 199.

[6] Porcari A, Fontana M, Gillmore JD. Transthyretin cardiac amyloidosis. Cardiovasc Res. 2023;118(18):3517–35. 35929637 10.1093/cvr/cvac119 PMC9897687

[7] Rubin J, Maurer MS. Cardiac amyloidosis: overlooked, underappreciated, and treatable. Annu Rev Med. 2020;71:203–19. 31986086 10.1146/annurev-med-052918-020140

[8] Kittleson MM, Ruberg FL, Ambardekar AV, Brannagan TH, Cheng RK, et al Writing Committee, . 2023 ACC Expert Consensus Decision Pathway on Comprehensive Multidisciplinary Care for the Patient With Cardiac Amyloidosis: A Report of the American College of Cardiology Solution Set Oversight Committee. J Am Coll Cardiol. 2023;81(11):1076–126. 36697326 10.1016/j.jacc.2022.11.022

[9] Ferrari Chen YF, Aimo A, Castiglione V, Chubuchna O, Morfino P, Fabiani I, et al. Etiological treatment of cardiac amyloidosis: standard of care and future directions. Curr Heart Fail Rep. 2025;22(1):16. doi: 10.1007/s11897-025-00701-4 40232627 PMC12000256

[10] Renger R, Renger J, Donaldson SI, Renger J, Hart G, Hawkins A. Comparing and contrasting a program versus system approach to evaluation: The example of a cardiac care system. 2020.

[11] Davies EL, Bulto LN, Walsh A, Pollock D, Langton VM, Laing RE, et al. Reporting and conducting patient journey mapping research in healthcare: a scoping review. J Adv Nurs. 2023;79(1):83–100. doi: 10.1111/jan.15479 36330555 PMC10099758

[12] Bartch VM, Vetting Wolf TL, Lee SA, Poncelet SA, Nemec SL, Morgenthaler TI. A service blueprint approach to prioritize operational improvements in a new outpatient clinic. Healthc (Amst). 2023;11(4):100715. doi: 10.1016/j.hjdsi.2023.100715 37748214

[13] Antonacci G, Lennox L, Barlow J, Evans L, Reed J. Process mapping in healthcare: a systematic review. BMC Health Serv Res. 2021;21(1):342. doi: 10.1186/s12913-021-06254-1 33853610 PMC8048073

[14] Kowalski CP, Nevedal AL, Finley EP, Young JP, Lewinski AA, Midboe AM, et al. Planning for and Assessing Rigor in Rapid Qualitative Analysis (PARRQA): a consensus-based framework for designing, conducting, and reporting. Implement Sci. 2024;19(1):71. doi: 10.1186/s13012-024-01397-1 39394597 PMC11468362

[15] Averill JB. Matrix analysis as a complementary analytic strategy in qualitative inquiry. Qual Inq. 2008;14:1–10. 10.1177/104973230201200611 12109729

[16] McGrath C, Palmgren PJ, Liljedahl M. Twelve tips for conducting qualitative research interviews. Med Teach. 2019;41(9):1002–6. doi: 10.1080/0142159X.2018.1497149 30261797

[17] Haukås Å, Tishakov T. Sharing interview questions in advance: methodological considerations in applied linguistics research. Eur J Appl Linguist. 2024;12(1):54–68.

