# Supplementary material for: Testing the utility of the first step of system evaluation theory in creating a system map of care for cardiac amyloidosis early detection: A case study
Source: PLoS One. 2026 Jan 16;21(1):e0339063. doi: 10.1371/journal.pone.0339063 (PMC12810811; doi:10.1371/journal.pone.0339063)
Supplement: S1 File — These questions were distributed to all workshop invitees to generate an exhaustive list of system components. (DOCX) [file pone.0339063.s001.docx]

**Supplement 1. Pre-workshop Brainstorming Questions**

- In addition to the patient and cardiologist, who else plays a role in the early diagnosis of cardiac amyloidosis?
- For each person named, what is their role in the early diagnosis of cardiac amyloidosis?
- What is important for each person named (in the above question) to increase the early diagnosis of cardiac amyloidosis (e.g., information, tools, resources, equipment)?
- How would each person best obtain what they need to increase the early diagnosis of cardiac amyloidosis? Please specify when and where (context for what they need) if possible.
- What are the barriers to obtaining what is needed for each person in the early diagnosis of cardiac amyloidosis?
- What is helpful in obtaining what is needed in the early diagnosis of cardiac amyloidosis?
