# Supplementary material for: Testing the utility of the first step of system evaluation theory in creating a system map of care for cardiac amyloidosis early detection: A case study
Source: PLoS One. 2026 Jan 16;21(1):e0339063. doi: 10.1371/journal.pone.0339063 (PMC12810811; doi:10.1371/journal.pone.0339063)
Supplement: S2 File — List of questions and processes used during the workshop to generate components of the system map. (DOCX) [file pone.0339063.s002.docx]

| **Supplement 2: Workshop Discussion Guide** |
| --- |
| **Thank you all for being here. As a brief reminder, we are going to record our discussion today. As you all know, despite advances in the early diagnosis and treatment of cardiac amyloidosis, we still need to develop implementation strategies to improve early detection of CA. The first step is to create a systems map that will help identify factors that help or hinder early cardiac amyloidosis early detection. That is why we invited all of you here to this workshop. You are all experts in different aspects of cardiac amyloidosis. We want to compile your various perspectives about how best to approach this disease.** |
| Here’s what we want you all to do for the breakout group discussion. We are asking each group to answer these questions:   - Who is involved, and what do they need to increase the early diagnosis of cardiac amyloidosis? - What are the barriers to the early diagnosis of cardiac amyloidosis? - What are the facilitators of increasing the early diagnosis of cardiac amyloidosis?   We have allotted 20 minutes for the group discussion. You are free to use the notepads and Post-it notes. Please designate one member to summarize the group’s discussion. After 20 minutes, we will take a 10-minute break, and then ask everyone to come back together to discuss as a large group. |
| Thank you for the discussion. We would now like to welcome the group spokesperson to report back on the highlights of your discussion. Please feel free to refer to the notepads if you used them. (Two facilitators begin mapping the responses on a whiteboard.) |
| After hearing everyone's input, what is missing? For each question, do you have something else to add   - Who else plays a role? - Is there anyone else? - What is each of these individuals' role?   - Any changes so far?   - Any additions? |
| What do each of these people need to complete their role?   - equipment/ tools, - information, - communication mechanisms.   - Anything else involved? |
| How are these individuals getting what they need? |
| How are these individuals working together? |
| Is there anything else that should be on this map that may have happened earlier in time or outside the space that we’ve already considered? |
| Where does early diagnosis begin? |
| Where does it end? |
| **Thank you all for your time. We will follow up with you after we have finalized our results.** |
