# Supplementary material for: Testing the utility of the first step of system evaluation theory in creating a system map of care for cardiac amyloidosis early detection: A case study
Source: PLoS One. 2026 Jan 16;21(1):e0339063. doi: 10.1371/journal.pone.0339063 (PMC12810811; doi:10.1371/journal.pone.0339063)
Supplement: S3 File — Questions asked of workshop participants who agreed to be interviewed to obtain their input on the completeness and accuracy of the post-workshop system map. (DOCX) [file pone.0339063.s003.docx]

**Supplement 3: Follow-up interview guide (member-checking)**

**Thank you for taking the time to evaluate the draft systems map for the early detection of cardiac amyloidosis. We took information from the workshop we conducted in January 2023 to create this draft map. We want to get your feedback on how we can improve this map.**

- What is missing from this map?
- How would you add/change to this map?
- Who else plays a role?

- Is there anything else that should be on this map that may happen earlier in time or outside the space \we have on this map?
- Where are there opportunities for improvement?
- Are there any other strategies you recommend to promote the timely screening for CA?

**Thank you all for your time. We will update this map according to your feedback.**
